# Metabolic Diseases—A Challenge for Public Health in the 21st Century

**DOI:** 10.3390/ijerph20186789

**Published:** 2023-09-20

**Authors:** Anna Garus-Pakowska

**Affiliations:** Department of Nutrition and Epidemiology, Medical University of Łódź, 90-419 Łódź, Poland; anna.garus-pakowska@umed.lodz.pl

Metabolic diseases refer to a broad term that includes all diseases that result from disturbances in the body’s biochemical metabolism.

The most common division concerns the cause of the disease. According to this, we distinguish between inherited and acquired metabolic diseases. Inherited metabolic diseases are associated with genetic disorders that affect the functioning of certain metabolic pathways. These conditions are relatively rare; examples include phenylketonuria, Gaucher disease, and mucopolysaccharidoses [1].

Acquired metabolic diseases are much more common, and their development is influenced by many factors. Most of us have encountered them in everyday life—these are known diseases such as obesity, osteoporosis, and diabetes. Over the last decades, the incidence of many of these diseases has increased dramatically, becoming a significant epidemiological problem. It is estimated that one third of the world’s population is overweight or obese [2]. In turn, every fifth woman (23.1%) and every tenth man (11.7%) in the world suffers from osteoporosis [3]. In 2017, approximately 462 million people had type 2 diabetes, constituting 6.28% of the world’s population (the prevalence rate was 6059 cases per 100,000) [4]. It is estimated that one in three adult Americans has Metabolic Syndrome [5]. These are just a few examples showing how metabolic diseases are a global problem.

These diseases are caused by broadly understood civilization changes and changes in lifestyle, including both a decrease in physical activity and changes in eating behavior (an improper diet, excessively high energy supply in relation to one’s needs, and the increased consumption of fast food, processed food, and various types of stimulants).

Many areas of human biology must be incorporated into the treatment of metabolic diseases. This will allow us to take a closer look at the causes and course of these diseases and to improve the treatment process by increasing the spectrum of impacts on the patient. One of the fields of science useful for the treatment of metabolic diseases is dietetics. Healthy (rational) nutrition is an element of a healthy lifestyle; it allows one to reduce their risk of many diseases, e.g., obesity and diabetes. However, the scope of its influence on the patient cannot be limited to the design and implementation of increasingly effective diets alone. It must interact with other therapeutic methods, primarily pharmacotherapy.

Many authors also emphasize the impact of physical activity exercises on the health of the body. Regular physical activity can help to reduce weight, reduce blood pressure, and improve lipid disorders, including raising HDL and lowering triglycerides [6].

Therefore, it can be concluded that the basis for the treatment of metabolic diseases is a radical lifestyle change (reducing body weight, following a proper diet, increasing physical activity).

We invite authors to submit manuscripts emphasizing the importance of proper nutrition and physical activity in health promotion and disease prevention. Publications on the epidemiology and risk factors of metabolic diseases, which are a challenge for public health in the modern world, are also of interest.
